# Endoplasmic Reticulum–Plasma Membrane Contact Sites: Regulators, Mechanisms, and Physiological Functions

**DOI:** 10.3389/fcell.2021.627700

**Published:** 2021-02-04

**Authors:** Chenlu Li, Tiantian Qian, Ruyue He, Chun Wan, Yinghui Liu, Haijia Yu

**Affiliations:** ^1^Jiangsu Key Laboratory for Molecular and Medical Biotechnology, College of Life Sciences, Nanjing Normal University, Nanjing, China; ^2^Department of Molecular, Cellular and Developmental Biology, University of Colorado, Boulder, CO, United States

**Keywords:** membrane contact sites (MCSs), endoplasmic reticulum (ER), plasma membrane, tether, lipid transfer, enzyme

## Abstract

The endoplasmic reticulum (ER) forms direct membrane contact sites with the plasma membrane (PM) in eukaryotic cells. These ER-PM contact sites play essential roles in lipid homeostasis, ion dynamics, and cell signaling, which are carried out by protein-protein or protein-lipid interactions. Distinct tethering factors dynamically control the architecture of ER-PM junctions in response to intracellular signals or external stimuli. The physiological roles of ER-PM contact sites are dependent on a variety of regulators that individually or cooperatively perform functions in diverse cellular processes. This review focuses on proteins functioning at ER-PM contact sites and highlights the recent progress in their mechanisms and physiological roles.

## Introduction

Intracellular trafficking between membrane-bound organelles is divided into two types, vesicular trafficking and non-vesicular trafficking. Vesicular trafficking is the predominant pathway to transport macromolecular substances and exchange information between organelles. The cargo is wrapped by or integrated into the membrane to form a vesicle, and exchange proteins or lipids between organelles through membrane fusion (Bonifacino and Glick, 2004; Südhof and Rothman, 2009). However, recent studies demonstrated that non-vesicular trafficking is another critical trafficking approach among intracellular membranous organelles, directly communicating through a close gap (typically within 10–30 nm) formed by two opposed membranes (Wong et al., 2019). This kind of intracellular communication is ensured by particular regions within the cell, defined as membrane contact sites (MCSs), structures mediated by protein-protein or protein-lipid interactions.

The largest membrane-bound organelle in eukaryotic cells is the endoplasmic reticulum (ER). It is the primary place for the synthesis of proteins and lipids, which are needed to maintain and propagate other membranous organelles and plasma membrane (PM) (Bonifacino and Glick, 2004). The ER extends throughout the whole cell and engages in broad communications with PM and other organelles by MCSs. ER-PM contact sites were first observed in muscle cells in the 1950s (Porter and Palade, 1957), and later were demonstrated as a general feature in eukaryotes. The MCSs formed between the ER and the PM provide an ideal platform for non-vesicular transport of lipids, ions, and many other signaling molecules (Gallo et al., 2016; Saheki and De Camilli, 2017a; Stefan, 2020). The architecture of ER-PM junctions is dynamically controlled by distinct tethering factors, and the cellular functions of ER-PM contact sites are highly dependent on those regulators located in these regions (Gallo et al., 2016). However, a variety of proteins localized at the crowded ER-PM junctions, frequently resulting in the co-existence of multiple regulators with similar or partially similar functions (Manford et al., 2012; Hoffmann et al., 2019; Johnson et al., 2019; Kang et al., 2019). They act synergistically to maintain the local microenvironment, which largely increases the difficulties of identifying their individual functions and mechanisms. Therefore, it is important to clarify how these proteins act in concert to play roles in the MCSs, especially under physiological or pathological conditions. This review focuses on the representative regulators localized at ER-PM contact sites, highlighting their physiological functions, molecular mechanisms as well as conservations in eukaryotes.

## Tethering Mechanisms of Proteins at ER-PM Contact Sites

**Figure 1:**
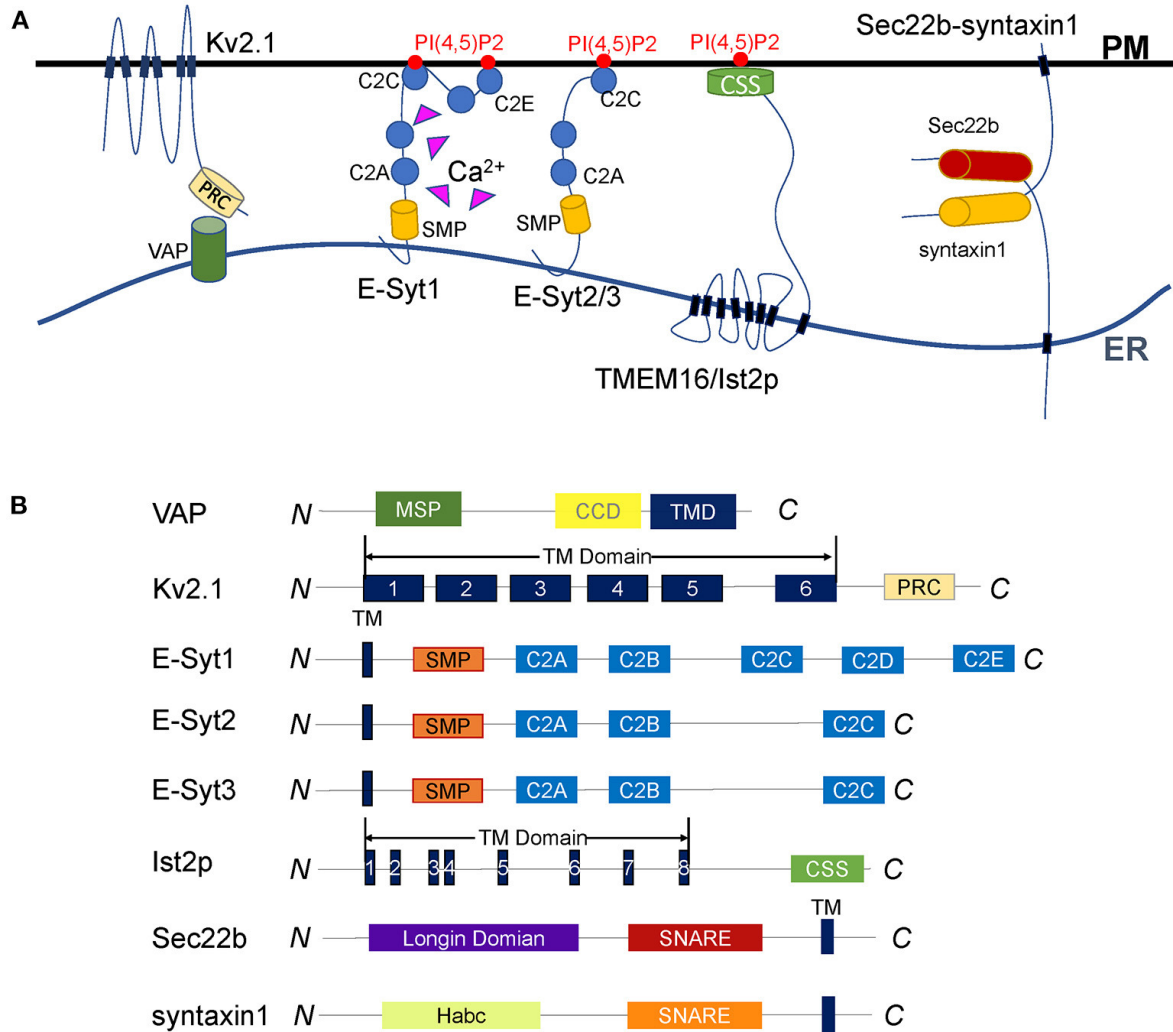
Diverse tethering mechanisms of proteins at ER-PM contact sites. **(A)** Representative illustration of the proteins that tether the ER and the PM. VAP anchors to the ER surface through the transmembrane domain and interacts with its binding partners to tether the ER and PM. The Kv2.1 channel is a VAP-binding partner. It anchors to the PM by six transmembrane domains and interacts with VAP through the C-terminal PRC domain. The Kv2.1-VAP interaction bridges the ER-PM junctions regulated by PRC domain phosphorylation. E-Syts are ER membrane proteins anchored to the ER *via* an N-terminal hydrophobic hairpin. E-Syt1 dynamically tethers the ER to the PM through a Ca^2+^-dependent interaction between the C2C domain and PI(4,5)P2. E-Syt2/3 constitutively maintains ER-PM junctions regardless of Ca^2+^. TMEM16/Ist2p is an eight-span integrin protein in the ER and connects the ER to the PM by its C-terminal CSS domain binding to PI(4,5)P2. The ER SNARE protein Sec22b forms an incomplete trans-SNARE complex with syntaxin1 on the PM that does not mediate membrane fusion but promotes the ER and PM tethering. **(B)** Diagrams of the membrane tethering proteins described in (A). The major functional domains are shown in each of the proteins.

The extensive cortical ER network is highly dynamic in eukaryotic cells. The tethering factors build and maintain the ER-PM contact sites demanded by diverse biological processes. Most proteins localized at the ER-PM contact sites can span and tether the two opposed membranes. It often happens that multiple proteins coordinate to tether the same MCSs and perform more than one physiological functions (Manford et al., 2012; Fernandez-Busnadiego et al., 2015; Kang et al., 2019). While some protein tethers are constitutively localized at ER-PM contact sites, the locations of others are dynamically regulated by stimuli such as calcium ions and phosphoinositides (Figure 1A) (Gallo et al., 2016; Okeke et al., 2016; Saheki and De Camilli, 2017a; Stefan, 2020). How these protein tethers accurately modulate the structure and plasticity of ER-PM contact sites has not been completely understood. Comprehensive understanding of the tethering mechanisms will shed important light on the dynamical control of the cortical ER network. In this section, we summarized the major ER-PM tethering proteins and discussed their membrane-targeting mechanisms.

### VAPs

Vesicle-associated membrane protein (VAMP)-associated protein (VAP) is an evolutionarily conserved ER membrane protein in all eukaryotes. It plays a vital role in many ER processes, especially in maintaining ER-PM contact sites. Loss of VAP by mutations leads to neurodegeneration, such as sporadic ALS or Parkinson's disease (Anagnostou et al., 2010; Kun-Rodrigues et al., 2015). There are mainly 2 VAPs (VAP-A and VAP-B) in mammals, 2 homologs (Scs2p and Scs22p) in yeast, and 10 homologs (VAP27-1 to VAP27-10) in *Arabidopsis*. VAP contains a major sperm protein (MSP) domain, a coiled-coil linker domain, and a C-terminal transmembrane domain required for ER surface location (Figure 1B) (Lev et al., 2008).

Although named by interaction with VAMP, VAP's primary function is not as a soluble *N*-ethylmaleimide-sensitive factor attachment protein receptor (SNARE) regulator. It binds more than 100 peripheral proteins, including those anchored into other organelles (Murphy and Levine, 2016). By interacting with those partners, VAP dynamically controls the junctions formed between the ER and other organelles such as Golgi, lipid droplets, mitochondria, endosomes, and PM (Murphy and Levine, 2016). By localization at particular contact sites, VAPs and their binding partners coordinate to mediate diverse cellular processes (Lev et al., 2008). However, the distribution of VAP in mammals is not limited to the MCSs but throughout the ER, suggesting it has other functions than membrane tethering. For example, VAP interacts with secernin-1 at the ER membrane to regulate dynamic ER remodeling (Lindhout et al., 2019).

Unlike in mammals, the homologs of VAPs in fission yeast and *Arabidopsis* are more concentrated at MCSs (Zhang et al., 2012; Wang et al., 2014). Scs2p and Scs22p were discovered as two major tethering factors at the ER-PM contact sites (Manford et al., 2012; Zhang et al., 2012). Scs2p was reported as an inositol binding protein that responds to phospholipid composition (Kagiwada and Hashimoto, 2007). However, there is no direct evidence showing Scs2p connects the ER to the PM through the interaction with phosphoinositides. In *Arabidopsis*, the tethering function of VAP27s at ER-PM contact sites was also identified. VAP27 co-localizes with NET3C and forms a tetra complex with microtubules and actin filaments to tether the ER to the PM (Wang et al., 2014). Recent studies suggested VAP27s-mediated ER-PM contact sites regulate plant endocytosis (Stefano et al., 2018; Wang et al., 2019). However, the detailed tethering mechanisms of VAP27s remain largely unknown.

The location of VAP largely relies on its binding partners. It is generally accepted that most of the VAP-mediated ER-PM tethering requires at least one PM-targeting partner. One primary class of the VAP-binding partners are cytoplasmic proteins containing an FFAT motif, which binds specifically to the MSP domain (Kamemura and Chihara, 2019). At MCSs, these FFAT-containing proteins interact with the PM through a membrane-targeting domain, for example, the pleckstrin homology domain (PHD). The lipid-transfer proteins (LTPs) are the most well-studied FFAT-containing proteins at MCSs. For instance, as an Scs2p/Scs22p-binding partner, yeast LTP Osh3p dynamically targets the PM by its PHD under the regulation of PM PI(4)P levels (Jansen et al., 2011; Kamemura and Chihara, 2019). Nir2, another type of FFAT-containing LTP, connects the PM by its C-terminal LNS2 domain binding to phosphatidic acid (PA) (Kim et al., 2013; Balla, 2018). The mechanisms of how these LTPs coordinate with VAP to regulate lipid metabolism will be discussed in the following corresponding section.

### Kv2 Channels

Voltage-gated potassium (Kv) channel is a tetramer composed of 4α subunits (70 kDa), and each subunit monomer contains six transmembrane helix segments (Figure 1B) (Christie, 1995; Yellen, 1998; Shah and Aizenman, 2014; Fu et al., 2017; Jedrychowska and Korzh, 2019). Kv2 channels Kv2.1 and Kv2.2, also named KCNB1 and KCNB2, are abundantly expressed in the brain and present in other tissues like muscle and pancreatic islets. The central part of the Kv2 channel is cytosolic, which forms large clusters in the ER-PM interface. The Kv2 is a delayed rectifier potassium channel, participates in the repolarization of neural action potentials (Murakoshi and Trimmer, 1999; Bishop et al., 2015). However, the clustered Kv2 channels do not readily conduct potassium (Lim et al., 2000; O'Connell et al., 2010), but involved in reshaping the ER-PM connections (Fox et al., 2015; Kirmiz et al., 2018a).

Kv2 channels are VAP-binding partners. They interact with VAPs through the C-terminal proximal restriction and clustering (PRC) domain (Lim et al., 2000; Johnson et al., 2019). This Kv2-VAP interaction mediates the ER-PM junctions responsible for Kv2 clustering (Johnson et al., 2018, 2019). The phosphorylation of serine residues in the PRC domain produces negative charges to enable VAP binding and control the clustering of Kv2 channels, which is the prerequisites for Kv2 channels-mediated ER-PM connections (Redman et al., 2007; Cobb et al., 2015; Johnson et al., 2018, 2019; Kirmiz et al., 2018b).

The clustering enables Kv2 channels to play a structural role in forming ER-PM junctions, and the non-conductive state is essential for avoiding electrically silencing neuronal activity (Fox et al., 2015). Hence, Kv2 clusters-induced ER-PM junctions could serve as a scaffold for other cell activities such as Ca^2+^ signaling and membrane trafficking. It has been reported Kv2.1 cluster promotes the coupling of PM L-type Ca^2+^ channels (LTCCs) and ER ryanodine receptor (RyR) Ca^2+^ release channels to generate partial Ca^2+^ release without the requirement of action potentials (Vierra et al., 2019). Recent studies revealed the Kv2.1 channels facilitate insulin exocytosis in pancreatic beta cells by their structural role of the clustering rather than the ability to conduct K^+^. Kv2.1 clusters could be applied as a target for insulin secretion (Fu et al., 2017; Greitzer-Antes et al., 2018). Currently, the localization mechanism of Kv2 channels at ER-PM junctions has been primarily uncovered, but the physiological function is still unclear.

### Extended Synaptotagmins (E-Syts)

E-Syts are integral membrane proteins anchored on the ER membrane. They are named by the similarity with synaptotagmins, key regulators in calcium-dependent vesicle fusion (Min et al., 2007). E-Syts are identified as a conserved family of tethering proteins at ER-PM contact sites. All E-Syts contain an N-terminal membrane anchor, followed by a synaptotagmin-like mitochondrial lipid-binding protein (SMP) domain and multiple C2 domains (Figure 1B) (Lee and Hong, 2006; Manford et al., 2012; Yu et al., 2016). While they are anchored to the ER membrane by the hydrophobic hairpin region, E-Syts can associate with the inner leaflet of the PM through their C-terminal C2 domains. The SMP domain is capable of harboring lipids, which we will discuss separately in the lipid exchange section.

The C2 domains are membrane-binding molecules representing a family of proteins with diverse functions (Rizo and Südhof, 1998). As for E-Syts, the C2 domains are connected in series to interact with acidic phospholipids on PM to mediate the ER-PM tethering. In mammals, E-Syt1 has five C2 domains, while E-Syt2 and E-Syt3 have three. The difference in numbers and characteristics of C2 domains among E-Syts leads to their distinct subcellular localization and tethering functions. E-Syt2 and E-Syt3 are located mainly at cortical ER. E-Syt1, by contrast, is broadly localized to the ER but migrate to ER-PM MCSs in response to elevated cytosolic Ca^2+^ (Min et al., 2007; Chang et al., 2013; Giordano et al., 2013; Idevall-Hagren et al., 2015). Cryo-ET studies indicated ER-PM contact sites mediated by E-Syts are structurally different from those bridged by STIM1 (Fernandez-Busnadiego et al., 2015). The average ER-PM distance at E-Syt3-mediated junctions is shorter than that observed at E-Syt1-mediated contact sites, although the latter could be shortened about 30% when cytosolic Ca^2+^ increases (Fernandez-Busnadiego et al., 2015). The C2C domain of E-Syt2/3 binds to PI(4,5)P2 through a conserved basic patch to constitutively maintain ER-PM contact sites. In contrast, the C2C domain of E-Syt1 interacts with PI(4,5)P2 upon Ca^2+^ binding to dynamically control the MCSs (Giordano et al., 2013; Idevall-Hagren et al., 2015; Saheki et al., 2016; Yu et al., 2016). In addition to ER-PM connections, E-Syts are also involved in the tethering of peroxisome-ER membrane contacts. They regulate cholesterol transport employing a similar C2C domain-PI(4,5)P2-binding mechanism (Xiao et al., 2019).

In yeast, the homologs of E-Syts are called tricalbins (Tcb1, Tcb2, and Tcb3) (Creutz et al., 2004; Schulz and Creutz, 2004; Lee and Hong, 2006). All the three tricalbins are major tethering contributors for ER-PM contact sites (Manford et al., 2012; Toulmay and Prinz, 2012). Recent studies showed tricalbins form curved cortical ER membrane with a requirement of C2 domains (Collado et al., 2019; Hoffmann et al., 2019). However, the detailed molecular mechanism of tricalbins in maintaining MCSs is still missing.

Plant SYT1 (synaptotagmin 1), the homolog of E-Syts in *Arabidopsis*, is enriched at ER-PM contact sites, especially the MCSs between immobile ER tubules and the PM (Yamazaki et al., 2010; Perez-Sancho et al., 2015; Ishikawa et al., 2018). The cortical ER network maintained by SYT1 correlates with the C2 domains, identical to E-Syts in mammals and tricalbins in yeast (Yamazaki et al., 2010). A recent study discovered that ionic stress could increase SYT1-mediated ER-PM connectivity by promoting the accumulation of PI(4,5)P2 on PM (Lee et al., 2019). These data suggest that the interaction between negatively charged lipids on PM and the C2 domains represents an evolutionarily conserved mechanism for E-Syt family proteins.

### Ist2p

Ist2p is the yeast homolog of the TMEM16, an eight-span integrin in the ER. The structure of Ist2p contains a specific ion channel followed by a long cytoplasmic C-terminal region riches in lysine and histidine residues (Figure 1B) (Juschke et al., 2005; Maass et al., 2009; Brach et al., 2011). The cortical localization of Ist2p relies on its C-terminal region, which was defined as the cortical sorting signal (CSS) (Brach et al., 2011). The CSS fragment regulates Ist2p expression and transports the protein to the PM, where it interacts with PI(4,5)P2 to bring the cortical ER and the PM closer to 15–50 nm (Juschke et al., 2005; Fischer et al., 2009; Maass et al., 2009; Wolf et al., 2012). The loss of Ist2p leads to an increase in the distance between the ER and the PM, suggesting Ist2p is a determinant for the span of ER-PM connections (Ercan et al., 2009).

The sorting mechanism of Ist2p from the ER to PM-associated domains is somewhat similar to the recruitment of STIM. They both bind to phospholipids on PM through the C-terminal domain, suggesting the recruitment of integral membrane proteins to PM through specific protein-lipid interactions represents a common mechanism. Ist2p was reported to be associated with the H^+^ pump Pma1 in the PM, allowing cells to adapt to different growth stages (Wolf et al., 2012). A recent study showed Ist2p and the LTP Osh6p are co-localized at ER-PM connections. Ist2p interacts with Osh6p to target the latter to the ER-PM contact sites, and they jointly participate in the lipid transport between the ER and the PM (D'Ambrosio et al., 2020).

While the function of Ist2p in yeast has been extensively studied, we currently still know little about its mammalian homolog TMEM16 (Hartzell et al., 2009). The two isoforms TMEM16A and TMEM16B, have recently been identified as calcium-activated chloride channels (Ercan et al., 2009; Xiao et al., 2011). However, whether TMEM16 and Ist2p have conserved functions at ER-PM contact sites remains to be clarified (Kunzelmann et al., 2016).

### Sec22b-Syntaxin1

SNARE proteins represent a superfamily in which the members share a conserved SNARE motif with about 60–70 residues. They are the core engine of intracellular vesicle fusion. SNAREs can be classified as Q-SNAREs and R-SNAREs. Membrane fusion is initiated when one R-SNARE on the vesicle pairs with three t-SNAREs on the target membrane to form a four-helix trans-SNARE complex (Sutton et al., 1998; Weber et al., 1998). Sec22 has three isoforms in mammals as Sec22a, Sec22b, and Sec22c. Only Sec22b has a SNARE motif and is conserved in yeast (Sun et al., 2020). Sec22b belongs to the R-SNARE family. It anchored to ER through a C-terminal transmembrane domain right after the SNARE motif. In addition to the coiled-coil SNARE motif and transmembrane domain, Sec22b contains an N-terminal longin domain conserved with a profilin-like folded structure (Figure 1B) (Fasshauer, 2003; Hong, 2005; Jahn and Scheller, 2006). The SNARE motif and longin domain of Sec22b may play essential roles in vesicular transport between the ER and the Golgi apparatus. While the SNARE motif forms four helixes with its cognate t-SNAREs, the longin domain regulates the membrane fusion by interaction with the SNARE motif (Daste et al., 2015).

Distinct to its traditional function on membrane fusion, Sec22b has another non-fusogenic role in PM expansion (Petkovic et al., 2014). It can interact with syntaxin1 to form a partial but tight SNARE complex, which could not drive the membrane fusion due to the absence of SNAP25. However, this kind of non-fusogenic SNARE bridge tethers the ER to the PM, and more interesting, this tethering function is conserved in yeast. The yeast Sec22p and Sso1p (the homolog of syntaxin1) interact with Osh2p and Osh3p to regulate non-vesicular lipid transport between the ER and the PM. The existence of these SNARE-mediated junctions can shorten the distances and improve the efficiency of lipid transport (Prinz, 2010; Petkovic et al., 2014). In the mammalian nervous system, one latest research found the Sec22b-syntaxin1 complex can interact with E-Syts and form a ternary complex that plays a vital role in PM expansion and axon growth (Gallo et al., 2020). Together, the Sec22b-syntaxin1 complex plays a role in the tethering of the ER to the PM, from which it indirectly participate in the regulation of lipid metabolism and contribute to PM extension and other physiological processes (Petkovic et al., 2014; Gallo et al., 2016).

### Versatile Tethering Regulators

Besides these representative tethering factors, there are many other versatile regulators at ER-PM contact sites. These regulators tether the ER and the PM when they perform their critical cellular functions, for example, the Ca^2+^ dynamics regulator stromal interaction molecule 1 (STIM1). Interestingly, STIM1-mediated membrane tethering is Ca^2+^-dependent. In response to the low concentration of Ca^2+^, The ER-anchored STIM1 oligomerizes and recognizes the PM polyphosphoinositides and Orai1 (Liou et al., 2007; Zhou et al., 2013). This action coordinatively regulates the membrane tethering and Ca^2+^ homeostasis. Another type of versatile-tethering protein is LTPs, which couple the membrane tethering and lipid metabolisms. Most LTPs anchor the ER through the transmembrane domain or VAP interactions while target the PM using protein-lipid interactions (Kim et al., 2015; Ghai et al., 2017; Naito et al., 2019). The mechanisms of these regulators will be discussed in the following sections.

Together, a variety of regulators have the ability of membrane tethering at ER-PM contact sites. They bridge the two membranes *via* diverse connections. Some connections are constant to maintain the primary cortical ER network, while the others are dynamically regulated to perform demanded functions. As the foundation of ER-PM contacts, all these tethering molecules coordinate to modulate the cellular processes through the fine tune of MCSs.

## Regulation of Ca^2+^ Dynamics at ER-PM Contact Sites

**Figure 2:**
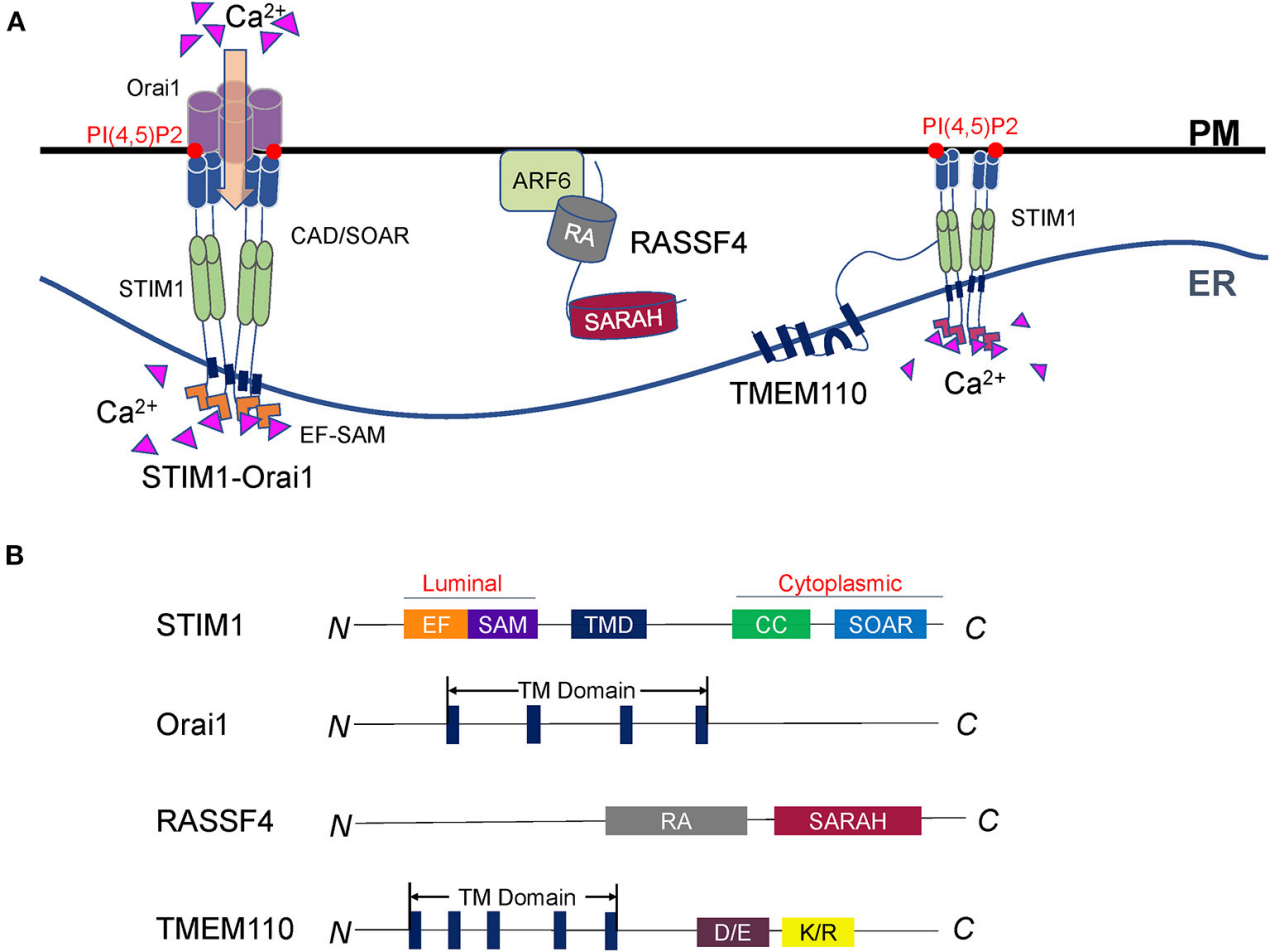
Regulation of Ca^2+^ dynamics at ER-PM contact sites. **(A)** Representative illustration of the STIM-Orai complex and its regulators. STIM1 in the ER and Orai1 on the PM compose the core machinery of SOCE. STIM1 senses the Ca^2+^ change in the ER lumen and forms oligomers. The STIM1 oligomers bind to polyphosphoinositides and activate Orai1 to import extracellular Ca^2+^. RASSF4 regulates SOCE and ER-PM junction through the control of the PM PI(4,5)P2 level, which is essential for the localization of STIM1. TMEM110 is an ER membrane protein that physically interacts with STIM1 to reshape ER-PM connections and facilitate STIM1 conformational conversion. Future studies are required to discover more STIM interacting partners, which is important to understand STIM proteins' sensing and coupling mechanisms. **(B)** Diagrams of the SOCE regulators described in A. The major functional domains are shown in each of the proteins.

As an important second messenger, Ca^2+^ is essential for many cellular and physiological processes, including gene transcription, protein modification, lipid metabolism, cell growth, and apoptosis (Stathopulos et al., 2006; Soboloff et al., 2012). So that precise and dynamic controls are needed to ensure calcium ions play proper functions at a specific time or place (Stathopulos et al., 2006). The cytoplasmic Ca^2+^ signals are generated by releasing Ca^2+^ from the calcium pool or the extracellular Ca^2+^ influx. The store-operated calcium entry (SOCE), a concept proposed in the 1990s, is a ubiquitous Ca^2+^ influx pathway at the ER-PM contact sites (Putney, 1986, 1990; Patterson et al., 1999; Yao et al., 1999). The Ca^2+^ entry is triggered when Ca^2+^ stores in the ER lumen depleted and the cytosolic Ca^2+^ concentration is at a low level. STIM proteins and Orai channels (Figure 2A) are the foundation proteins in the regulation of SOCE and Ca^2+^ signals (Liou et al., 2005; Roos et al., 2005; Feske et al., 2006; Vig et al., 2006; Zhang et al., 2006).

### STIM-Orai Complexes

STIM crosses the ER membrane and senses Ca^2+^ in the cavity of the ER (Williams et al., 2001; Zhang et al., 2005; Feske et al., 2006). Orai is a Ca^2+^ release-activated Ca^2+^ channel on PM (Chakrabarti and Chakrabarti, 2006; Prakriya et al., 2006; Soboloff et al., 2006a). Upon Ca^2+^ depletion, STIM protein interacts with Orai and initiates SOCE (Carrasco and Meyer, 2011; Zhou et al., 2013; Balla, 2018). There are two STIM proteins in mammals: STIM1 and STIM2. Both of them are mainly located at the ER with a similar structure (Hogan and Rao, 2015). STIM is anchored to the ER *via* a transmembrane domain, with an N-terminal domain in the ER lumen and a C-terminal domain in the cytoplasm (Figure 2B). The expression pattern of STIM1 and STIM2 are different. In most tissues, the expression level of STIM1 is higher than STIM2 in support that STIM1 is the predominant STIM protein that regulates the influx of Ca^2+^ in non-excitable cells (Soboloff et al., 2006b; Collins and Meyer, 2011; Hogan and Rao, 2015; Prakriya and Lewis, 2015).

STIM has the EF-hand and stereo alpha motif (SAM) domains in the lumen of the ER. Its cytoplasmic side contains several coiled-coil domains and a CRAC activation domain (CAD, also known as the STIM-Orai activation region, Or SOAR) (Manji et al., 2000; Yang et al., 2012; Prakriya and Lewis, 2015). In the presence of Ca^2+^, the EF-hand domains are tightly bound to the SAM domain. Therefore, the STIM protein exists as a monomer and in an inactive state, in which the nearby coiled-coil domain blocks CAD/SOAR. When the ER calcium pool is exhausted, the STIM protein senses Ca^2+^ change (Yuan et al., 2009). The structure of the EF-SAM domain becomes loose and stretched. STIM proteins dimerize from the ER lumen side to the cytoplasm and further form oligomers. These activated STIM1 oligomers interact with PI(4,5)P2 and the Orai channel through the released CAD/SOAR domain to accurately mediate the Ca^2+^ influx (Park et al., 2009; Ma et al., 2015; Prakriya and Lewis, 2015).

Although occupying a similar structure, the EF-SAM domains from STIM1 and STIM2 show different affinity to Ca^2+^. STIM1 is in a state of autoinhibition at rest but highly active upon Ca^2+^ depletion. STIM2 is more sensitive to tiny changes in the ER calcium store due to its low Ca^2+^ affinity, thus could be activated in response to less Ca^2+^ change (Zheng et al., 2011; Soboloff et al., 2012; Hogan and Rao, 2015). The different biophysical characteristics of STIM1 and STIM2 enable cells to sense changes in intracellular Ca^2+^ concentration accurately and take further actions (Brandman et al., 2007; Prakriya and Lewis, 2015). It seems reasonable that the basal Ca^2+^ homeostasis maintenance at rest is the primary responsibility of STIM2 (Wang et al., 2009; Kar et al., 2012). However, no significant change of Ca^2+^ level was observed in the calcium store of STIM2 KO cells at rest. In some tissues, for example, the neuronal cells and dendritic cells, the expression of STIM2 is significantly higher than STIM1, suggesting STIM2 may have other functions than the regulation of basal Ca^2+^ homeostasis (Williams et al., 2001; Oh-Hora et al., 2008; Prakriya and Lewis, 2015).

Many proteins around the STIM-Orai complex participate in SOCE. It is worth noted that SOCE recruits E-Syt1 to ER-PM junctions and rearranges adjacent ER structures into circular MCSs, which in turn stabilizes STIM-Orai clusters and accelerates Ca^2+^ replenishment (Kang et al., 2019). Another recent study showed ER protein Anoctamin 8 (ANO8) is translocated to STIM1-Orai1-mediated contact sites in a PI(4,5)P2-dependent manner. ANO8 further recruits the ER-localized SERCA (Sarco/endoplasmic reticulum Ca^2+^-ATPase) Ca^2+^ pump to replenish the Ca^2+^ reservoir, which may inactivate SOCE and regulate the receptor-stimulated Ca^2+^ signaling (Jha et al., 2019; Stefan, 2020).

### RASSF4

The RAS association domain family (RASSF) consists of 10 members (RASSF1-10) localized at the cytoplasmic side of the PM. RASSF4 contains a C-terminal RAS association (RA) domain linked to a Sav-RASSF-Hpo (SARAH) domain (Figure 2B) (Chan et al., 2013; Iwasa et al., 2013). While the RA domain mediates the interactions with RAS GTPases, the SARAH domain was reported to facilitate dimerization between SARAH domain-containing proteins (Chan et al., 2013).

At ER-PM contact sites, RASSF4 acts in concert with ARF6, the upstream regulator of type I phosphatidylinositol phosphokinase (PIP5K), to regulate PI(4,5)P2 levels on the PM (Chen et al., 2017). Since PI(4,5)P2 is essential to position STIM1 and E-Syts, RASSF4 participates in the regulation of SOCE and ER-PM junctions indirectly through the regulation of PI(4,5)P2 homeostasis (Dickson, 2017).

### TMEM110

ER-resident transmembrane protein 110 (TMEM110) is a STIM-activating enhancer (STIMATE). It contains 4–5 transmembrane domains, co-localized with STIM (Figure 2B). TMEM110 can remodel the short-term physiological junctions and relocate STIM1. Overexpression of TMEM110 leads to the formation of large STIM aggregates, while knockdown of this gene reduces STIM1 puncta at the ER-PM junctions (Jing et al., 2015; Quintana et al., 2015).

Furthermore, TMEM110 could physically interact with STIM1 and interfere with the autoinhibition of CAD/SOAR. When the Ca^2+^ storage is exhausted, STIM1 converts its conformation, facilitating the TMEM110 C-terminus interaction with the coiled-coil domain of STIM1. It releases the autoinhibition of CAD/SOAR and activates Ca^2+^ channel Orai (Hooper and Soboloff, 2015; Jing et al., 2015). Overall, TMEM110 is an ER protein that cooperates with STIM to reshape ER-PM connections and regulate calcium signaling dynamically. These studies indicate the STIM-Orai signaling heavily relies on proteins that regulate the ER-PM connections or the STIM conformation.

## Enzymes at ER-PM Contact Sites

**Figure 3:**
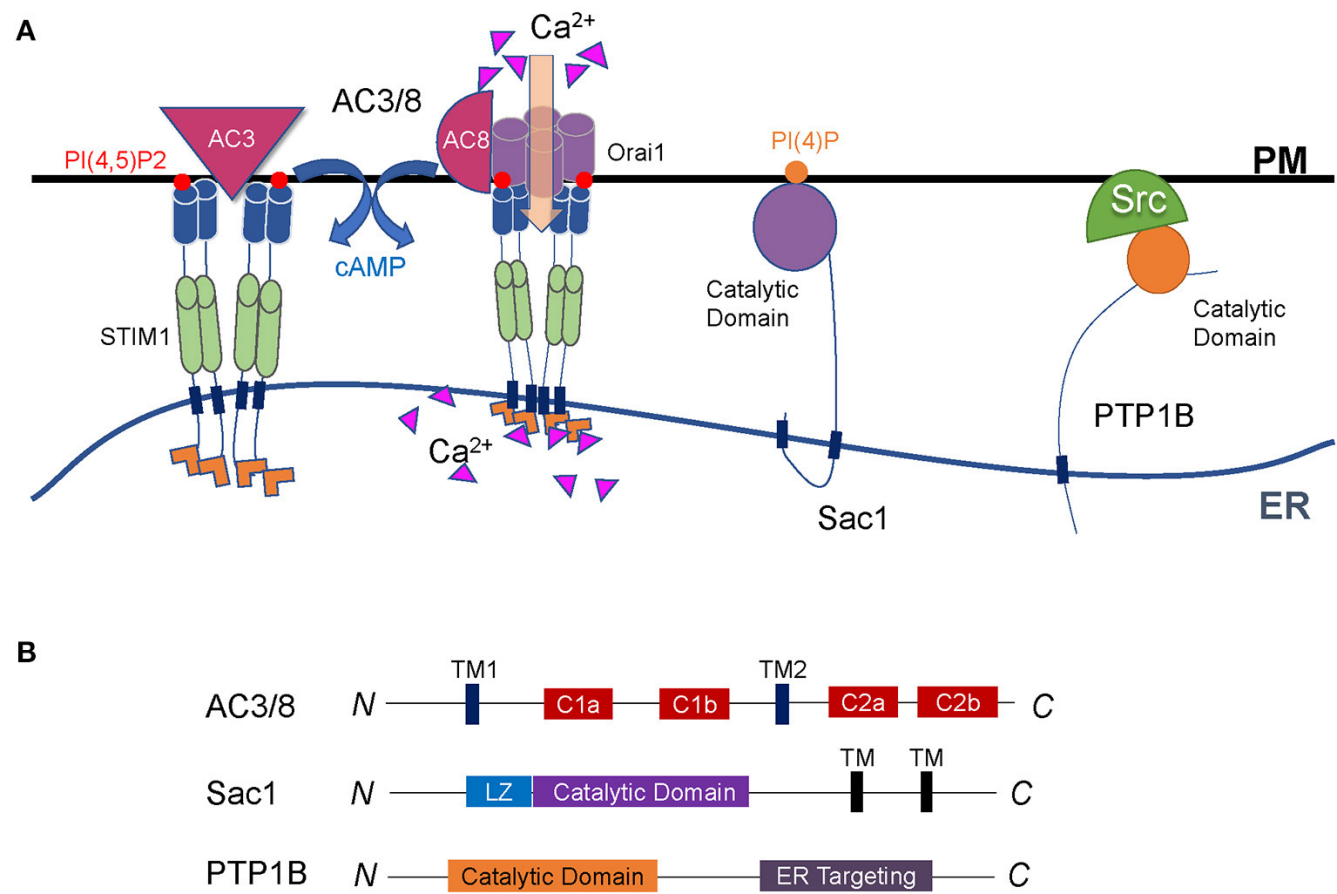
Regulation of cell signaling by enzymes at ER-PM contact sites. **(A)** Representative illustration of enzymes functioning at the interface between the ER and the PM. Both AC3 and AC8 are located on the PM through multiple transmembrane domains. AC3 interacts with STIM1, and AC8 binds directly to Orai1. They are both regulated by Ca^2+^ signals to produce cAMP. Sac1 is a conserved PI(4)P phosphatase anchored to the ER and can dephosphorylate PI(4)P both on the PM and in the ER membrane. PTP1B is anchored to the ER membrane *via* a C-terminal fragment and dephosphorylates its substrates on the PM through the cytosolic catalytic domain. **(B)** Diagrams of the enzymes described in A. The major functional domains are shown in each of the proteins.

MCSs are ideal platforms to exchange molecules and local signals between organelles, dependent on their carriers or enzymes. Many protein enzymes, especially the phosphatases, play critical regulatory roles in ER-PM contact sites (Figure 3A) (Saheki and De Camilli, 2017a). They can catalyze substrates either in *cis* or in *trans* to participate in the regulatory network of many cellular processes such as cyclic adenosine 3′,5′-adenosine monophosphate (cAMP) signaling, calcium dynamics, and lipid metabolism.

### AC3/8

Adenylate cyclase (AC) is an important signaling molecule downstream of G protein-coupled receptors. It is located on the PM *via* two multi-transmembrane domains and contains two catalytic domains (Figure 3B) (Cooper et al., 1995; Cooper and Crossthwaite, 2006; Dessauer et al., 2017). AC regulates cAMP, thereby participating in various physiological processes. Nine AC subtypes have been identified in mammals. Among them, the AC3 and AC8 are located at ER-PM junctions, and both of their activities are regulated by Ca^2+^. AC3 regulates blood glucose homeostasis, which makes it a new target for the development of anti-obesity drugs. However, AC8 plays a crucial role in neuroplasticity rather than in glucose regulation (Zachariou et al., 2008; Bogard et al., 2014; Wu et al., 2016).

Inside the cell, many signals transmit between the ER and the PM. In addition to the SOCE-regulated Ca^2+^ signal, cAMP is another vital signal which usually functions as a second messenger. At the ER-PM junctions, AC3 is an enzyme that relies on STIM1. STIM1 interacts with AC3 and generates cAMP, and this process is called storage operational cAMP signaling (SOcAMPS) (Lefkimmiatis et al., 2009; Maiellaro et al., 2012; Willoughby et al., 2012). AC8 directly interacts with Orai1 to alter the Ca^2+^ microenvironment under the PM, which in turn activates AC8 and produces cAMP (Willoughby et al., 2010, 2012). The dynamic interaction between Ca^2+^ and cAMP signals at the ER-PM junctions represents an important scenario of cell homeostasis and plays vital roles in physiology and pathology (Lefkimmiatis et al., 2009; Maiellaro et al., 2012; Willoughby et al., 2012; Okeke et al., 2016).

### Sac1

Localized on the ER and Golgi apparatus, suppressor of actin 1 (Sac1) is a phosphoinositide phosphatase whose protein sequence and function are both highly conserved in yeast and mammals. In mammals, Sac1 protein commonly expresses in adult and embryonic tissues (Del Bel and Brill, 2018). The C-terminal region anchored Sac1 to the ER. A conserved catalytic CX5R (T/S) motif in the N-terminal domain enables Sac1 to have a catalytic function (Figure 3B) (Manford et al., 2010; Saheki and De Camilli, 2017a). The role of Sac1 is to remove phosphoric acid from the inositol ring to balance the level of PI(4)P (Del Bel and Brill, 2018).

Primarily as a PI(4)P phosphatase between the ER and the PM, Sac1 was proposed to either act in *trans* on the opposed PM PI(4)P or act in *cis* on the ER PI(4)P (Manford et al., 2010; Stefan et al., 2011; Mesmin et al., 2013). In the latter, ORP5/8 (Osh6p/7p in yeast) transfers PM PI(4)P to the ER, where Sac1 dephosphorylates the lipid on the same ER membrane. Sac1-catalyzed PI(4)P hydrolysis is essential to maintain PI(4)P concentration gradient, which facilitates the continuous exchange of PI(4)P/PS between the ER and the PM (Chung et al., 2015; Moser von Filseck et al., 2015b; Del Bel and Brill, 2018). Therefore, Sac1 indirectly controls the lipid metabolism where the ER and the PM are in close contact.

However, PI(4)P alone is insufficient to localize Sac1 to the ER-PM contact sites. Other proteins that connect the ER and the PM may jointly participate in the Sac1 localization. For example, the activated SOCE increases the amount of Sac1 in contact with the PM, while disruption of E-Syt2-mediated ER-PM junctions reduces the access of Sac1 on the PM (Dickson et al., 2016). Sac1 was found co-localized with E-Syt2 at ER-PM contact sites. E-Syt2 narrows ER and PM distance, which may restrict Sac1 to the right position. Sac1 consumes PI(4)P in this microdomain and thus produces the PI(4)P gradient (Dickson et al., 2016). However, it is still uncertain whether E-Syt2 directly interacts with Sac1.

### PTP1B

Protein tyrosine phosphatase 1B (PTP1B) is a non-receptor phosphatase and belongs to the PTP family. First isolated from the human placenta, PTP1B is anchored to the surface of the ER membrane *via* a C-terminal fragment composed of 35 proline-rich residues (Frangioni et al., 1992). The N-terminus of PTP1B protein contains the catalytic domain with two proline-rich motifs (Figure 3B). PTP1B plays a catalytic function at ER-PM junctions by dephosphorylation of its substrates located on the PM through the cytosolic catalytic domain (Anderie et al., 2007).

PTP1B has several identified substrates. These substrates have diverse functions that make PTP1B play various roles in cellular physiology. For example, ER-bound PTP1B dynamically interacts with the protein tyrosine kinase Src on the PM, controls Src activation, and recruits adhesion complexes (Monteleone et al., 2012). PTP1B also plays roles in tumor growth, metastasis, and metabolism. It has double-sided effects with either promoting or suppressing cancer in tumor tissues, depending on the active substrate and cell environment (Lessard et al., 2010). Exploring the roles of PTP1B in individual tumors will provide new ideas for the diagnosis and treatment of tumors. The insulin signaling pathway and glucose metabolism is another process that PTP1B negatively regulates. The tyrosine-phosphorylated insulin receptor, insulin receptor substrate-1, and AKT are all possible targets of PTP1B (Abdelsalam et al., 2019). Targeting PTP1B is considered a strategy to treat insulin resistance and type 2 diabetes by improving insulin sensitivity (Hussain et al., 2019).

## Lipid Exchanges at ER-PM Contact Sites

**Figure 4:**
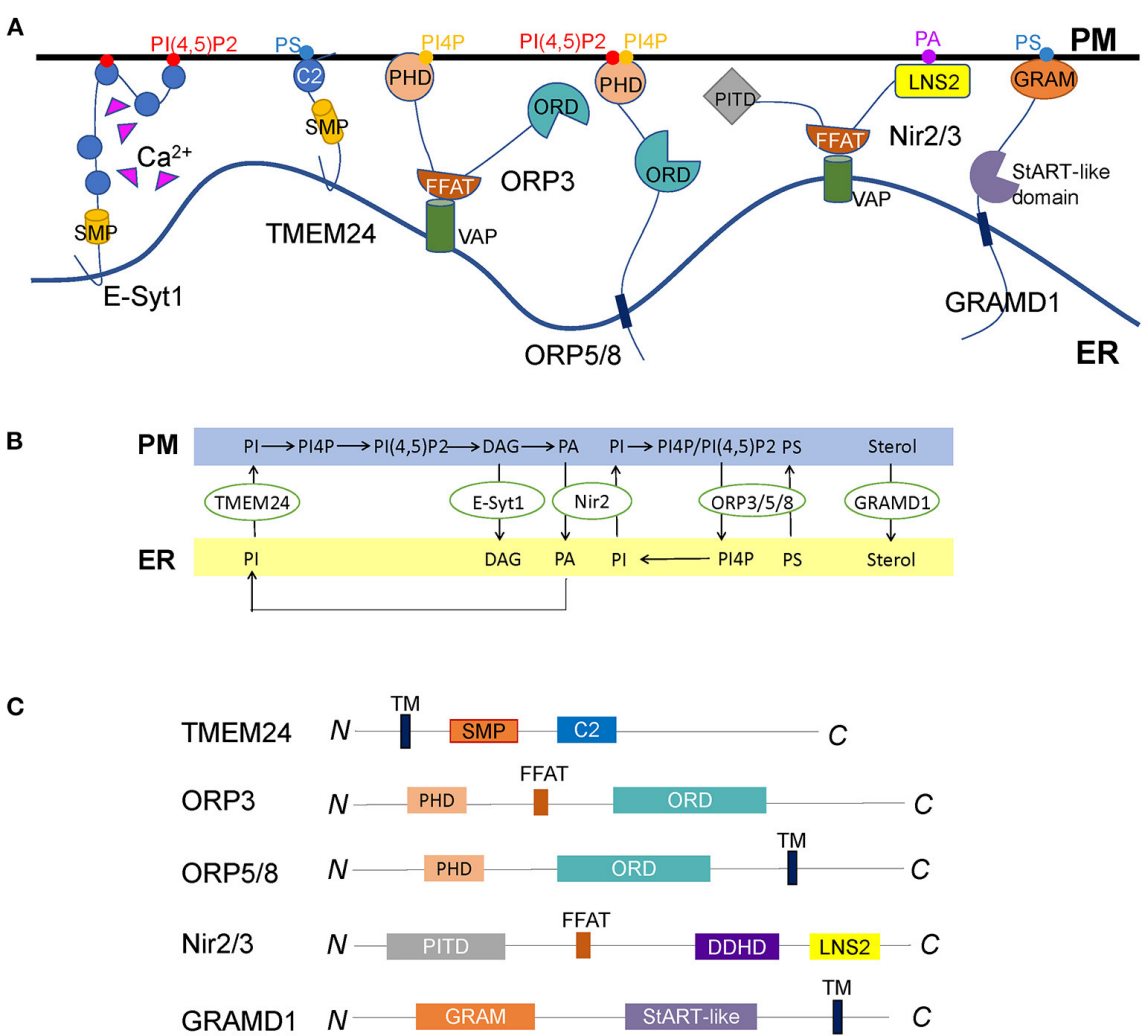
Diverse LTPs functioning at the ER-PM contact sites. **(A)** Representative illustration of LTPs located at the ER-PM junctions. E-Syt1 transfers phospholipids and DAG directly through the SMP domain between the ER and the PM, regulated by interactions of the C2 domains with Ca^2+^. TMEM24 is another SMP domain protein localized to the ER through a transmembrane domain. It transfers PI from the ER to the PM controlled by the dephosphorylation of the C-terminal polybasic region. Whether other SMP domain proteins, for example, E-Syt2/3 and tricalbins, mediate lipid transport at ER-PM contact sites remains unknown. ORP3, ORP5, and ORP8 are so far discovered ORPs at ER-PM contact sites. While ORP5 and ORP8 are ER membrane proteins, ORP3 is anchored to the ER through the interaction of its FFAT motif with VAP. All the three ORPs contain a PHD to interact with the PM lipids and an ORD to exchange PI(4)P/PI(4,5)P2 and PS between the ER and the PM. It remains elusive why the cell has three ORPs at ER-PM contact sites to mediate the same lipids. The discovery of the initial triggers of these LTPs might solve this problem. Nir2 and Nir3 are anchored to the ER *via* interactions of FFAT motifs with VAPs and connect to the PM by LNS2 domains, facilitating the PI and PA exchange between the two membranes. How other tethering molecules, such as E-Syts and Kv2s, couple with Nir2/3 to mediate PA/PI exchange need to be further explored. GRAMD1s are anchored to the ER *via* a C-terminal transmembrane domain and interact with cholesterol and PS on the PM through the N-terminal GRAM domain to connect the two opposed membranes. Thus, the accessible cholesterol is transported from the PM to the ER by StART-like domains. Whether there are other sterol transfer proteins transporting PM cholesterol to the ER or other organelles remains to be discovered. **(B)** The summary of LTPs-mediated lipid exchange at ER-PM contact sites. **(C)** Diagrams of the lipid transfer proteins described in A, except E-Syt1, are shown in Figure 1B. The major functional domains are shown in each of the proteins.

The ER is the central organelle that synthesizes various lipids such as phospholipids and cholesterol, which need to be transported to or exchanged with other organelles and PM. Unlike the bulk lipid transports mediated by vesicle fusion, LTPs are able to sense and transport particular lipids between organelles that are mostly happened within a short distance like at the MCS regions (Wong et al., 2019). The ER forms extensive membrane junctions with the PM where LTPs play vital roles in the regulation of lipid metabolism as well as other physiological processes (Figures 4A,B) (Kentala et al., 2016; Saheki and De Camilli, 2017a; Cockcroft and Raghu, 2018; Jeyasimman and Saheki, 2019; Stefan, 2020).

### SMP Domain Proteins

SMP domain proteins are evolutionarily conserved in eukaryotes. The SMP domain was first discovered in 2006 by the sequence analysis of a mitochondrial integral membrane protein. Later it was identified as a member of the superfamily of tubular Lipid-binding (TULIP) domain-containing proteins that have the ability to harbor lipids in the hydrophobic cavity (Kopec et al., 2010; Alva and Lupas, 2016). SMP domain proteins are commonly localized at MCSs formed by organelles and play versatile functions such as lipid transport, Ca^2+^ homeostasis, and signaling (Saheki and De Camilli, 2017a).

Benefited from the SMP domain, E-Syts are considered as LTPs at ER-PM contact sites (Giordano et al., 2013; Saheki and De Camilli, 2017b). The crystal structure of E-Syt2 showed SMP domain forms a dimer of approximately nine nm-long cylinders that harbors glycerolipids without selectivity (Schauder et al., 2014). E-Syt1, E-Syt-2, and E-Syt-3 have been demonstrated to form homo- and heterodimers inside the cell (Giordano et al., 2013). By the *in vitro* reconstituted system, E-Syt1 was identified as a Ca^2+^ dependent LTP, which directly transfers glycerophospholipids and DAG between the ER and the PM (Saheki et al., 2016; Yu et al., 2016). The SMP dimer is indispensable for E-Syt1 to transfer lipids. Considering the length of SMP dimer is far less than the average distance between the ER and the PM, the “shuttle model” in which SMP dimer shuttles between the ER and the PM to transport lipids is more acceptable. This shuttle model was further supported by an artificially designed assay using the DNA-origami nanostructures to define precise distances between membranes (Bian et al., 2019).

In addition to the SMP domain, the C2 domains in E-Syt1 are also essential to regulate lipid transfer (Saheki et al., 2016; Yu et al., 2016; Bian et al., 2018). At least, both the C2A and C2C domains are indispensable in E-Syt1-mediated lipid transport (Saheki et al., 2016; Yu et al., 2016; Bian et al., 2018). The C2C domain is the predominant region for membrane tethering, facilitating lipid transport by shortening the distance. The C2A domain was proposed as an autoinhibitory domain that inactivates the SMP domain. Ca^2+^ releases this autoinhibition to enable lipid transport (Bian et al., 2018). Recent studies in yeast demonstrated SMP domain and C2 domains of tricalbins act in concert to form highly curved ER peaks on the cortical ER membrane facing the PM, raising a possibility that the C2A domain involves in the interaction with the ER membrane to facilitate the lipid transport (Collado et al., 2019; Hoffmann et al., 2019).

Although the membrane tethering function of the E-Syt family is conserved in eukaryotes, it was unclear whether all family members are capable of transporting lipids between the ER and PM. Only E-Syt1 in mammalian cells was identified to mediate lipid exchange directly. Although the locations of E-Syt2 and E-Syt3 at ER-PM contact sites are not affected by Ca^2+^, Ca^2+^ does bind to their C2A domains and induce a local protein conformational transition (Xu et al., 2014). Whether E-Syt2 and E-Syt3 directly mediate lipid exchange are still open questions to the field.

As a highly conserved protein family, E-Syts likely play essential roles in cell physiology. Unexpectedly, they're non-essential proteins. No obvious defects were observed in E-Syts triple knockout mice (Sclip et al., 2016; Tremblay and Moss, 2016). Neither mammalian cells lacking E-Syts nor yeast cells lacking tricalbins showed significant abnormalities (Manford et al., 2012; Toulmay and Prinz, 2012; Saheki et al., 2016). Given that many factors coordinate at ER-PM junctions, one possible explanation could be the functional redundancy. Other factors may replace the function of E-Syts once the latter is omitted (Saheki, 2017). However, E-Syts and tricalbins do have physiological effects. For example, E-Syts can maintain PM lipid homeostasis, promote nerve transmission and synaptic growth, modulate virus-induced membrane fusion, mediate the endocytosis of FGFR, and play roles in insulin secretion and diet-induced obesity development (Saheki et al., 2016; Tremblay et al., 2016; Kikuma et al., 2017; El Kasmi et al., 2018; Xie et al., 2019; Nath et al., 2020; Zhang et al., 2020). Tricalbins act in maintaining PM integrity (Toulmay and Prinz, 2012; Collado et al., 2019). More interesting, plant SYT1 is required for withstanding mechanical stress, maintaining cell membrane integrity and virus movement, suggesting this protein family is of importance to cell physiology (Min et al., 2007; Chang et al., 2013; Giordano et al., 2013; Idevall-Hagren et al., 2015).

Transmembrane protein 24 (TMEM24) is another SMP domain protein localized at ER-PM contact sites. It is anchored to the ER membrane through an N-terminal transmembrane domain, followed by an SMP domain, a C2 domain, and a polybasic C-terminal region (Figure 4C). TMEM24 is involved in regulating insulin secretion and neuronal excitability (Pottekat et al., 2013; Lees et al., 2017; Sun et al., 2019). Like E-Syts, the SMP domain forms a dimer in TMEM24, and each SMP domain binds one lipid molecule, one less than that in E-Syt2 (Schauder et al., 2014; Lees et al., 2017). TMEM24 selectively transports PI from the ER to the PM, supplying PM with PI(4,5)P2 during signal transductions. This process is regulated by protein kinase C (PKC)-dependent phosphorylation of the C-terminal PM binding regions in response to cytosolic Ca^2+^ (Lees et al., 2017).

Glucose-stimulated insulin secretion is regulated by the inositol phosphate signaling pathway and Ca^2+^. While TMEM24 transfers the PI(4,5)P2 precursor PI from the ER to the PM, E-Syt1 clears the PI(4,5)P2 metabolite DAG on PM. Since the dissociation of TMEM24 from PM is controlled by PKC-mediated phosphorylation, and E-Syt1 regulates PKC activity, E-Syt1 is considered an indirect regulator of TMEM24 (Xie et al., 2019). Both of them play a role in phosphoinositide metabolism and Ca^2+^ homeostasis, thus indirectly regulate insulin secretion in pancreatic β cells (Xie et al., 2019).

### ORPs

The oxysterol-binding protein (OSBP) and its related proteins (ORPs) compose a conserved family that mediates non-vesicular lipid transports at the MCSs (Im et al., 2005; de Saint-Jean et al., 2011; Olkkonen and Li, 2013; Du et al., 2015). There are two conserved domains in this protein family, the PHD and OSBP-related domain (ORD). The PHD plays a role in membrane docking by interactions with anionic lipids such as phosphatidylserine (PS), PI(4)P, and PI(4,5)P2. The ORD is a ligand binding and lipid exchange domain (Kentala et al., 2016; Cockcroft and Raghu, 2018). Besides, ORPs have either an FFAT domain or a transmembrane domain through which they are located on the surface of membranous organelles, including the ER (Mesmin et al., 2013; Pulli et al., 2018).

ORP3, ORP5, and ORP8 are ORPs mainly localized at ER-PM contact sites in mammals. ORP5 and ORP8 possess a single C-terminal transmembrane domain that anchors them on the ER surface and a PHD to interact with the PM (Figure 4C). They were reported to mediate the PI(4)P/PS exchange cycle that transfers PS from the ER to the PM and move PI(4)P from the PM to the ER. Sac1 depletes PI(4)P and generates a PI(4)P gradient to drive this exchange (Chung et al., 2015; Moser von Filseck et al., 2015b; Dickson et al., 2016). However, another study showed PI(4,5)P2, rather than PI(4)P is the critical lipid for the targeting of ORP5 and ORP8 to the PM (Ghai et al., 2017). Besides ER-PM contact sites, ORP5 and ORP8 are also localized at other MCSs such as ER-mitochondria and ER-lipid droplet contact sites (Galmes et al., 2016; Du et al., 2020). Unlike ORP5 and ORP8, ORP3 is anchored to the ER *via* its FFAT motif that interacts with VAP protein (Figure 4C). Recent studies uncovered that ORP3 is capable of regulating PI(4)P homeostasis and Ca^2+^ dynamics by activating PKC (Dong et al., 2020; D'Souza et al., 2020; Gulyás et al., 2020).

In yeast, the conserved ORP family is called oxysterol-binding homology (Osh) protein. At ER-PM contact sites, Osh6p and Osh7p mediate the exchange of PI(4)P and PS fueled by PI(4)P metabolism, similar to the ORP5 and ORP8 in mammals (Maeda et al., 2013; Moser von Filseck et al., 2015a). Osh3p is more like ORP3 in mammals, which binds PI(4)P and recruits the Sac1p to ER-PM contact sites to regulate the PM PI(4)P levels (Stefan et al., 2011; Tong et al., 2013; Omnus et al., 2020). These studies demonstrated that phosphoinositides, mainly the PI(4)P and PI(4,5)P2, are common lipid ligands for the ORP family localized at ER-PM contact sites. Although 12 conserved members have been identified in *Arabidopsis*, the functions of ORPs in plant lipid metabolism are still poorly understood (Skirpan et al., 2006). Whether the functions and mechanisms of ORPs discovered in mammals and yeast are conserved in plants remain to be determined.

ORPs localized at ER-PM contact sites play multiple roles in cell physiology. For example, Osh2p and Osh3p interact with Myo5p and Scs2p to bridge the ER contact with endocytic areas and facilitate actin polymerization (Encinar Del Dedo et al., 2017). ORP3 participates in Ca^2+^ homeostasis and cell adhesion (Lehto et al., 2008). ORP5 and ORP8 can regulate cancer growth, making them potential drug targets for cancer therapy (Ishikawa et al., 2010; Guo et al., 2017).

### Nir2/3

Nir2 and Nir3 belong to the PI transfer protein (PITP) family, a class of central players involved in phospholipid homeostasis at ER-PM contact sites. They are the mammalian ortholog proteins of Drosophila retinal degeneration B (rdgB), which was proven to transfer PI and phosphatidylcholine (PC) between membrane bilayers (Amarilio et al., 2005). The structure of Nir2/3 contains a PI-transfer domain at the very N-terminus, then followed by an FFAT motif, six hydrophobic stretches, and a C-terminal LNS2 domain (Figure 4C). Nir2/3 is anchored to the ER through the binding of the FFAT sequence to the VAP protein and connects with PM by the interaction of the LNS2 domain with phosphatidic acid (PA) (Kim et al., 2013; Balla, 2018).

PA and PI are interconverted lipid second messengers that play roles in many signaling pathways, coupled by the Nir2/rdgB family. Phospholipase C (PLC) hydrolyses PI(4,5)P2 to generate DAG and its phosphorylated lipid PA. After binding PA, Nir2/rdgB is translocated to ER-PM contact sites to exchange PI and PA between the two opposed membranes (Chang et al., 2013; Kim et al., 2013, 2015; Balla, 2018). This process coordinates local lipid metabolism with downstream signaling at ER-PM contact sites (Kim et al., 2013). A recent study found that Nir2 and Kv2.1 are co-localized at ER-PM contact sites in neuronal cells, indicating Kv2-VAP tethers may regulate Nir2 localization and PI homeostasis (Kirmiz et al., 2019). Different from Nir2, the ability of Nir3 is to sense subtle PA production and sustain basal PM PI(4,5)P2 levels (Chang and Liou, 2015). They cooperatively regulate PI(4,5)P2 homeostasis at ER-PM contact sites.

### GRAMD

The protein containing the Glucosyltransferases, Rab-like GTPase activators, and myotubularins (GRAM) domain was named GRAMD, a recently discovered class of conserved ER proteins. Bioinformatics studies identified six GRAMD proteins (Ysp1p, Sip3p, Ysp2p, Lam4p, Lam5p, and Lam6p) in yeast and five members (GRAMD1a, GRAMD1b, GRAMD1c, GRAMD2, and GRAMD3) in mammals (Gatta et al., 2015). However, only three GRAMD1s in mammals contain StART-like lipid transfer domains. GRAMD2 and GRAMD3 are supposed not to mediate lipid transport (Naito et al., 2019). GRAMD proteins are anchored to ER *via* its C-terminal transmembrane domain and target the PM using the N-terminal GRAM domain (Figure 4C) (Stefan et al., 2011; Chu et al., 2015; Besprozvannaya et al., 2018).

The existence of the StART-like domain makes GRAMD1 proteins contribute to PM sterol homeostasis by recognizing accessible PM cholesterol and transporting it to the ER (Holthuis and Levine, 2005; van Meer et al., 2008; Sandhu et al., 2018). A recent study showed GRAMD1s form homo- and heteromeric complexes that interact with the free cholesterol and PS on the PM by the GRAM domain. Thus, the accessible cholesterol is transported to the ER through StART-like domains. Loss of the three GRAMD1s leads to a significant expansion of the available PM cholesterol pool, suggesting GRAMD1s are major cholesterol transporters between the ER and the PM (Sandhu et al., 2018; Naito et al., 2019). Different from GRAMD1, GRAMD2 co-localizes with E-Syts and help to maintain the ER-PM contacts. Based on this tethering function, GRAMD2 may play a role in SOCE and Ca^2+^ homeostasis by the recruitment of STIM1 (Besprozvannaya et al., 2018).

The trafficking of low-density-lipoprotein (LDL)-cholesterol is essential in cholesterol metabolism. Imbalance in this process could cause diseases, for example, the Niemann-Pick type C (NPC). The ER is the central organelle senses and synthesizes endogenous cholesterol. After endocytosis, the vast majority of LDL-cholesterol is transported to the PM (Pfisterer et al., 2016). The PM cholesterol could then supply the ER by GRAMD1s (Sandhu et al., 2018). However, about 30% of LDL-cholesterol is directly transported from endosomes/lysosomes to the ER, mediated by ORP1L and STARD3. When ER cholesterol is excessive, these sterol-transfer proteins can act in the opposite direction, transporting the cholesterol to the endosomes (Eden et al., 2016; Wilhelm et al., 2017). Recent studies indicate NPC1 tethers the ER to the endocytic organelles and facilitates cholesterol egress through ORP5 and GRAMD1s. Both of the LTPs localized at the ER-endocytic organelles MCSs in response to the cholesterol levels (Du et al., 2011; Höglinger et al., 2019).

CERT (ceramide transport protein) is another LTP containing a StART-like domain. In addition to glycerophospholipids and sterols, ceramide is also synthesized in the ER. CERT anchors to the ER *via* the FFAT motif-VAP interaction. It contains a PHD that interacts with PI(4)P to build the ER-Golgi contacts. Through the StART-like domain, CERT transports ceramide to the Golgi apparatus, where glucosylceramide and sphingomyelin are synthesized (Hanada et al., 2003). Glycolipid-transfer protein, another VAP-interacting LTP, transports glucosylceramide from *cis*-Golgi to *trans*-Golgi or the ER (Smith et al., 2006; Halter et al., 2007; Backman et al., 2018). These processes are indispensable for sphingomyelin and glycosphingolipid homeostasis (Breslow, 2013; Hanada, 2018).

### Coordination of Ca^2+^ Signaling and Phospholipid Metabolism

Many regulators coordinate to form an extensive protein network at ER-PM contact sites, regulating intracellular signal transductions coupled with Ca^2+^ and phospholipid signaling. The elevated cytoplasmic Ca^2+^ triggers the enrichment of E-Syt1 at ER-PM contact sites (Giordano et al., 2013). In another way, the ER-Ca^2+^ depletion induces STIM1 translocation to ER-PM contact sites (Liou et al., 2007). Both the Ca^2+^-regulated processes enhance the ER-PM connections, which subsequently promotes the recruitment of Nir2 to the ER-PM interface (Chang et al., 2013). Nir2 binds and transfers PA from the PM to the ER (Kim et al., 2015). In turn, it moves PI from the ER to the PM and generates PI(4)P and PI(4,5)P2, in which step RASSF4 and ARF6 participate (Stathopulos et al., 2006; Chen et al., 2017; Dickson, 2017). PI(4,5)P2 reinforces relocations of E-Syts and STIM1 at the contact sites and further regulates Ca^2+^ dynamics. The consumption of excess PI(4,5)P2 generates DAG and PA, which facilitate Nir2 enter the next circle. Overall, this network combines SOCE regulators with LTPs to develop a synergistic effect between Ca^2+^ signaling and PI(4,5)P2 metabolism at the ER-PM junctions and extend the duration of signal transductions (Dickson, 2017; Ong and Ambudkar, 2020).

## Conclusions

The ER communicates with the PM through direct physical contacts, which are regulated by various proteins. These key players cooperatively mediate the reactions between the opposed membranes and drive diverse fundamental cellular processes. ER-PM contact sites are involved in the regulation of ion and lipid transports, signal transductions, ER morphology and remodeling, membrane trafficking, and yeast polarized growth (Encinar Del Dedo et al., 2017; Ng et al., 2018, 2020; Kang et al., 2019; Kirmiz et al., 2019; Weber-Boyvat et al., 2020). Substantial progress has been made toward understanding their functions and mechanisms (Table 1) (Saheki and De Camilli, 2017a; Wang et al., 2017; Ong and Ambudkar, 2020; Stefan, 2020). However, all proteins located at the MCSs seem to tether the membranes. Multiple regulators perform the same functions at the ER-PM contact sites in some cellular processes (Manford et al., 2012; Collado et al., 2019). The unique significance of these proteins needs to be further explored. For example, why are three E-Syts in yeast and mammals but only one in plants and flies?

**Table 1:** Summary of the proteins functioned at ER-PM contact sites.

**Proteins**			**Locations**	**Comments**
**Mammalians**	**Yeast**	**Plants**		
VAP-A	Scs2p	VAP27-1 to-10	ER	VAPs are ER membrane proteins that dynamically tether the PM through its binding partners
VAP-B	Scs22p			
Kv2	—	—	PM	The clustered Kv2 channels reshape the ER-PM connections by the interaction with VAPs
E-Syt1	Tcb1	SYT1	ER	The C2 domains play essential roles in the tethering function of E-Syt protein family. E-Syt1 bidirectionally transfers phospholipids and DAG. Whether other E-Syt proteins directly transfer lipids remain unknown
E-Syt2	Tcb2			
E-Syt3	Tcb3			
TMEM16	Ist2p	—	ER	The interaction of Ist2p with PI(4,5)P2 brings the ER and the PM closer to 15-50 nm. Whether TMEM16 has similar functions remains unknown
Sec22b-syntaxin1	Sec22p-Sso1p	—	ER-PM	The two proteins form a non-fusogenic SNARE bridge between the opposed membranes
STIM1-Orai1	—	—	ER-PM	In SOCE, STIM1 senses the Ca^2+^ level and acts as a switch for Orai1 to import external Ca^2+^. The mechanism and physiological function of STIM1-Orai1 needs a further study
RASSF4	—	—	PM	RASSF4 is a regulator of PI(4,5)P2 homeostasis that further regulates SOCE
TMEM110	—	—	ER	TMEM110 is a STIM-activating enhancer
AC3/8	—	—	PM	AC3 and AC8 interact with STIM1 and Orai1 separately to produce cAMP
Sac1	—	—	ER	Sac1 dephosphorylates PI(4)P to regulate phosphoinositide metabolism
PTP1B	—	—	ER	PTP1B dephosphorylates its substrates on the PM, and plays roles in glucose metabolism, and other physiological processes
ORP3	Osh2p	ORPs	ER	ORPs located at ER-PM contact sites mediate the exchange of PI(4)P/PI(4,5)P2 with PS, their individual mechanism needs to be further studied. The functions of ORPs in plants are still not clear
ORP5	Osh3p			
ORP8	Osh6p			
	Osh7p			
TMEM24	—	—	ER	TMEM24 transfers PI from the ER to the PM
Nir2	—	—	ER	Nir2 and Nir3 exchange PI and PA between the ER and the PM
Nir3				
GRAMD1s	—	—	ER	GRAMD1s contribute to PM sterol homeostasis

Given that most of the regulators at ER-PM contact sites are highly conserved, it seems unlikely the cell keeps redundant proteins at this narrow and crowded place during evolution. These proteins are more likely of great significance to the cell. However, the general functional redundancy gives us trouble understanding their exact physiological functions. One speculation is each of these functional redundant proteins still has its features. Take tethering as an example. Although all protein tethers are capable of bridging the ER and the PM, the ER-PM junctions formed by distinct tethers have variable architectures and different mechanisms (Petkovic et al., 2014; Fernandez-Busnadiego et al., 2015; Johnson et al., 2018). These differences make them feasible to regulate the diverse cellular processes or play roles in specific conditions such as ER stress or cell damage. The same situation also happens in LTPs. It remains unclear why multiple LTPs transfer one specific lipid at MCSs. Perhaps the future discovery of their regulation and triggering mechanisms will give us the answer.

Tissue specificity is another possibility. Proteins with similar functions at ER-PM contact sites may individually play a predominant role in the specific type of cells, depending on their expression enrichments or stimuli. Studies of those proteins in specific tissues will be a way to identify their physiological roles. Recent studies have already confirmed this possibility (Guo et al., 2017; Kirmiz et al., 2018a; Zhang et al., 2020). On the other hand, some regulators' functions are not related to ER-PM contact sites (Tremblay et al., 2016; El Kasmi et al., 2018). Further efforts are needed to study their correlations.

To better understand the functions and mechanisms of ER-PM contact sites, high-resolution structures of protein-membrane complexes mimicking MCSs or partially mimicking MCSs will be expected. The development of advanced Cryo-EM and live-cell imaging technology will enable us to comprehensively understand the protein network-mediated ER-PM contact sites. Many regulators at these sites are implicated in disease pathologies. Studies of these proteins for their physiological and pathological functions will be of great significance for understanding the disease occurrence, new drug developments, and clinical applications.

## Author Contributions

CL, TQ, RH, CW, YL, and HY wrote the manuscript. All authors contributed to the article and approved the submitted version.

## Conflict of Interest

The authors declare that the research was conducted in the absence of any commercial or financial relationships that could be construed as a potential conflict of interest.
